# Waist-to-height ratio and metabolic phenotype compared to the Matsuda index for the prediction of insulin resistance

**DOI:** 10.1038/s41598-021-87266-z

**Published:** 2021-04-15

**Authors:** Katharina Lechner, Benjamin Lechner, Alexander Crispin, Peter E. H. Schwarz, Helene von Bibra

**Affiliations:** 1 Kardiologie, Deutsches Herzzentrum München, Technische Universität München, Munich, Germany; 2 Department of Internal Medicine IV, Ludwig-Maximilians-Universität München, Munich, Germany; 3 Institute for Medical Information Processing, Biometry and Epidemiology, Ludwig-Maximilians-Universität München, Munich, Germany; 4 Center for Evidence-Based Healthcare, University Hospital Carl Gustav Carus, TU, Dresden, Germany; 5 Paul Langerhans Institute Dresden of the Helmholtz Center Munich at University Hospital and Faculty of Medicine, TU Dresden, Dresden, Germany; 6 German Center for Diabetes Research (DZD E.V.), Neuherberg, Germany; 7 Technical University of Munich, Stelznerstr. 7, 81479 Munich, Germany

**Keywords:** Endocrine system and metabolic diseases, Preventive medicine

## Abstract

Current screening algorithms for type 2 diabetes (T2D) rely on fasting plasma glucose (FPG) and/or HbA1c. This fails to identify a sizeable subgroup of individuals in early stages of metabolic dysregulation who are at high risk for developing diabetes or cardiovascular disease. The Matsuda index, a combination of parameters derived from a fasting and postprandial insulin assay, is an early biomarker for metabolic dysregulation (i.e. insulin resistance/compensatory hyperinsulinemia). The aim of this analysis was to compare four widely available anthropometric and biochemical markers indicative of this condition [waist-to-height ratio (WHtR), hypertriglyceridemic-waist phenotype (HTW), triglycerides-to-HDL-C ratio (TG/HDL-C) and FPG] to the Matsuda index. This cross-sectional analysis included 2231 individuals with normal fasting glucose (NFG, n = 1333), impaired fasting glucose (IFG, n = 599) and T2D (n = 299) from an outpatient diabetes clinic in Germany and thus extended a prior analysis from our group done on the first two subgroups. We analyzed correlations of the Matsuda index with WHtR, HTW, TG/HDL-C and FPG and their predictive accuracies by correlation and logistic regression analyses and receiver operating characteristics. In the entire group and in NFG, IFG and T2D, the best associations were observed between the Matsuda index and the WHtR (r = − 0.458), followed by HTW phenotype (r = − 0.438). As for prediction accuracy, WHtR was superior to HTW, TG/HDL-C and FPG in the entire group (AUC 0.801) and NFG, IFG and T2D. A multivariable risk score for the prediction of insulin resistance was tested and demonstrated an area under the ROC curve of 0.765 for WHtR and its interaction with sex as predictor controlled by age and sex. The predictive power increased to 0.845 when FPG and TG/HDL-C were included. Using as a comparator the Matsuda index, WHtR, compared to HTW, TG/HDL-C and FPG, showed the best predictive value for detecting metabolic dysregulation. We conclude that WHtR, a widely available anthropometric index, could refine phenotypic screening for insulin resistance/hyperinsulinemia. This may ameliorate early identification of individuals who are candidates for appropriate therapeutic interventions aimed at addressing the twin epidemic of metabolic and cardiovascular disease in settings where more extended testing such as insulin assays are not feasible.

## Introduction

Type 2 diabetes (T2D) has reached pandemic proportions worldwide and constitutes one of the largest threats to healthcare systems globally^1^. Its global costs are considerable and are projected to increase further^2^. In the United States, more than half of the adult population live with pre-diabetes or diabetes^3^. Beyond imposing a substantial economic burden to societies globally, diabetes and its clinical complications are leading causes for reduced health span and premature mortality^1^. Early identification is crucial to avoid and/or delay the onset of diabetes and its clinical macro- and microvascular complications^1,3^.

Of concern, the reliance on biomarkers indicative of elevated blood glucose levels such as fasting plasma glucose (FPG)^4^ and 2-h postload plasma glucose (2hPG) measurements during an oral glucose tolerance test (OGTT)^5^ results in missed opportunities for early diagnosis of diabetes^6^.

Assessing glucose at several time points (0, 30, and 120 min) during an OGTT ameliorates the prediction of diabetes^7,8^, the association with cardiovascular disease and mortality^9–11^ and the prediction for the risk of future diabetes and all-cause mortality^6^. The prediction of diabetes and pre-diabetes can further be refined by addition of fasting and, in particular, postprandial insulin measurements^7,12^.

Insulin resistance and compensatory hyperinsulinemia are a common metabolic abnormality characterizing individuals with various cardiometabolic risk factors such as T2D, hypertension, dyslipidemia and central adiposity. These conditions combine to significantly elevate the risk for numerous diseases including atherosclerotic cardiovascular disease (ASCVD)^13–15^. Impaired insulin action is a major underlying feature of these clinical conditions. There is an unmet need for techniques which allow the assessment of insulin sensitivity in clinical settings for the prevention of T2D and ASCVD^12^. The Matsuda index is an index of whole-body insulin sensitivity derived from the simultaneous assessment of insulin and glucose levels during an oral glucose tolerance test (OGTT) with five measurement at 0, 30, 60, 90 and 120 min. This index represents a composite of both hepatic and peripheral tissue sensitivity to insulin and considers insulin sensitivity in the basal state and after the ingestion of a defined glucose load^16^. It is correlated strongly (r = 0.73, P < 0.0001) with the euglycemic insulin clamp which is a direct measure of insulin sensitivity. The Matsuda index has been shown to be superior to the HOMA-IR in detecting risk and reclassifying insulin resistance^17^. It has thus been suggested as the best surrogate for the hyperinsulinemic-euglycemic glucose clamp technique^16^ which is not feasible for routine clinical application. Collectively, the Matsuda index, which can be calculated from glucose and insulin measurements derived from an OGTT, is an effective clinical tool to define insulin sensitivity (i.e. the ability of tissues to respond to the signal of insulin) and secretory defects in individuals with impaired glucose homeostasis^16^.

It is however worth noting that fasting and postprandial insulin assays are not widely available in primary care settings. In this regard, clinical indices based on anthropometric and/or biochemical markers indicative of truncal adiposity and atherogenic dyslipidemia have been suggested as markers for early stages of metabolic derangement^15^. These indices include the waist-to-height ratio (WHtR)^18,19^, the hypertriglyceridemic-waist phenotype (HTW)^20,21^ and the triglycerides-to-HDL-C ratio (TG/HDL-C)^22,23^. They have been suggested as markers for insulin resistance and/or hyperinsulinemia long before changes in blood glucose levels accrue^15^, and are associated with atherosclerotic plaque phenotype^24–26^ and cardiovascular risk^27^.

The aim of this data-driven analysis was to compare the predictive value of indices indicative of metabolic dysregulation (i.e. insulin resistance/compensatory hyperinsulinemia) to FPG using as a comparator the Matsuda index.

## Methods

### Subjects and methods

This paper is a re-analysis of cross-sectional data acquired from a cohort of 2231 individuals (> 20 years of age) from an outpatient diabetes clinic in the city of Dresden and adjacent areas in Germany over a period of 17 years from 1996 to 2012^28,29^. Participants without a previous history of T2D were recruited via newspaper advertisements and received a financial incentive for their participation.

As assessed by FPG, participants were categorized into three groups: 1333 with normal (< 5.6 mmol/L) fasting glucose (NFG), 599 with impaired (5.6–6.9 mmol/L) fasting glucose (IFG) and 299 with T2D (≥ 7.0 mmol/L). Baseline characteristics of the groups are depicted in Table 1.

**Table 1:** Characteristics of all participants: differences between the subgroups are *p* < 0.001 unless indicated otherwise.

	All	NFG	IFG	Diabetes
Number (%)	2231 (100)	1333 (59)	599 (26)	299 (13)
Men nr (%)	1004 (45.0)	520 (39.0)	311 (51.9)	173 (57.9) *ns*
Age (years)	56 ± 14	53 ± 15	59 ± 12	61 ± 11 *ns*
BMI (kg/m^2^)	27.5 ± 4.7	26.5 ± 4.3	28.5 ± 4.8	29.9 ± 5.2
Waist (cm)	95 ± 13	92 ± 13	98 ± 12	104 ± 12
WHtR	0.56 ± 0.08	0.54 ± 0.07	0.58 ± 0.07	0.61 ± 0.008
SBP (mmHg)	132 ± 18	129 ± 16	137 ± 18	143 ± 20
DBP (mmHg)	82 ± 12	79 ± 11	84 ± 11	87 ± 12
Total cholesterol (mmol/l)	5.5 ± 1.1	5.4 ± 1.0	5.6 ± 1.1**	5.7 ± 1.4 *ns*
LDL-C (mmol/l)	3.3 ± 1.0	3.3 ± 1.0	3.5 ± 1.0	3.3 ± 1.0 ns#
Triglycerides (mmol/l)	1.5 ± 1.1	1.3 ± 0.7	1.5 ± 0.8	2.3 ± 2.1
HDL-C (mmol/l)	1.5 ± 0.4	1.6 ± 0.4	1.5 ± 0.4	1.3 ± 0.4
TG/HDL-C ratio	1.2 ± 1.3	0.9 ± 0.7	1.2 ± 0.9	2.2 ± 2.8
Fasting blood glucose (mmol/l)	5.7 ± 1.3	5.0 ± 0.4	6.0 ± 0.3	8.4 ± 1.8
Fasting insulin (pmol/l)	80 ± 50	67 ± 41	90 ± 46	117 ± 69
HOMA IR	3.0 ± 2.4	2.1 ± 1.3	3.4 ± 1.8	6.1 ± 4.0
Matsuda index	4.7 ± 3.0	5.7 ± 3.2	3.4 ± 1.8	2.3 ± 1.2
WHtR (class 0–2)	0.56 ± 0.50	0.45 ± 0.49	0.67 ± 0.47	0.79 ± 0.40
HTW phenotype (class 1–3)	1.7 ± 1.0	1.5 ± 1.1	2.0 ± 0.9	2.3 ± 0.8

Data are given as mean ± standard deviation. In comparison to NFG ns = no significance, *p < .05 and **p < 0.01. In comparison to IFG *ns* = *no significance*, #*p* < *0.05* and ##*p* < *0.01.*

*NFG* normal fasting glucose, *IFG* impaired fasting glucose, *SBP* systolic blood pressure, *DBP* diastolic blood pressure.

Exclusion criteria were acute and chronic medical conditions with a strong impact on metabolism and life expectancy and pharmacotherapy with drugs known to interfere with glucose metabolism.

The screening protocol has been published before^28,29^ and included measurements of heart rate, waist circumference, weight and height which were obtained by trained medical staff according to standard operating procedures. Blood pressure was measured twice in supine position after a 5-min resting period using a mercury sphygmomanometer. Fasting lipid and glucometabolic state were assessed following an overnight fasting period (≥ 10 h). Plasma glucose and insulin were measured before and after ingestion of 75 g glucose at 30-min intervals for 2 h. Insulin sensitivity was assessed by a 5-point OGTT [Matsuda index = ISI(comp)]^16^:

g = glucose, i = insulin, 0 = fasting; 30, 60, 90, 120 = minutes after a glucose challenge with 75 g glucose.

$$ {\text{ISI(comp) = }}\frac{10000}{{\sqrt {g_{0} \times i_{0} \times \frac{{(g_{0} \cdot 15 + g_{30} \cdot 30 + g_{60} \cdot 30 + g_{90} \cdot 30 + g_{120} \cdot 15}}{120}} \times \frac{{(i_{0} \cdot 15 + i_{30} \cdot 30 + i_{60} \cdot 30 + i_{90} \cdot 30 + i_{120} \cdot 15}}{120}}} $$

Suggested cut-off values for defining insulin resistance by the Matsuda index are inconsistent across the literature, ranging from a cut off of < 2.5 in the original publication^16^ to < 3.5^30^, to < 4.3^31^ up to over 6.4^17^. Based on the diverging literature on different cut-off values for the Matsuda index, we defined a Matsuda cut-off of ≤ 4.0 as a clinically reasonable value to differentiate between individuals with and without insulin resistance in our Caucasian cohort. It should be noted that ROC analysis is not limited by the specific choice of a cut-off value, thereby the chosen cut-off value for the Matsuda index per se is not directly relevant to the main findings reported in this paper.

In order to assign severity grades to the screening parameter WHtR, a normal ratio was considered if WHtR ≤ 0.5^18^, risk if WHtR > 0.5 to ≤ 0.6 and abnormal if WHtR > 0.6. The respective subgroups of the HTW phenotype were defined as: normal HTW (waist circumference for men (women) < 90 cm (< 85) and triglycerides < 2.0 mmol/L); risk HTW (waist circumference for men (women) ≥ 90 cm (≥ 85 cm) and triglycerides < 2.0 mmol/L or triglycerides ≥ 2.0 mmol/L and waist circumference for men (women) < 90 cm (< 85 cm) and abnormal HTW (waist circumference ≥ 90 cm (≥ 85 cm) and triglycerides ≥ 2.0 mmol/L)^28^.

Plasma glucose was measured by the hexokinase method [interassay coefficient of variation (CV) 1.5%]. Insulin levels were measured by enzyme immunoassay (BioSource EUROPE, Nivelles, Belgium, interassay CV 7.5%). Triglyceride levels were measured by an enzymatic assay (Boehringer Mannheim, Mannheim, Germany) and HDL-C by precipitation with dextran sulfate (Boehringer Mannheim).

The study was approved by the local ethics committee, Ethik-Kommission, Medizinische Fakultät Carl Gustav Carus, Technische Universität Dresden. Written informed consent was obtained from all participants according to the guidelines of the institutional review boards for human studies at the Technical University of Dresden.

### Statistical analysis

SPSS Statistics for Windows version 17.0 (SPSS Inc., Chicago, IL, USA) was used for statistical analysis. Additional logistic regression analyses were performed using the Statistical Analysis System SAS 9.4 for Windows (SAS Institute, Cary, NC, USA). Data are presented as mean ± standard deviation. Group comparisons were performed by Anova and followed by Bonferroni posthoc tests in the three subgroups. WHtR, HTW, TG/HDL-C and FPG were evaluated as the four surrogate estimates of metabolic derangement/insulin resistance. Their relations with the Matsuda index were analyzed by correlation analysis and their discriminatory power by receiver operating characteristics (ROC) curves. Multivariable risk scores for the prediction of insulin resistance were developed using Firth logistic regression models. Models were fitted in a random sample comprising two thirds of the patient records. The remaining third was used as validation sample. Model discrimination and calibration were assessed using ROC and calibration curves. All tests are two-sided on an alpha level of 5%.

## Results

Clinical and laboratory characteristics indicative of metabolic dysregulation incrementally increased from the NFG to the IFG and the T2D subgroup and so did the classification by screening tools (Table 1). HDL-C decreased as expected. In the three subgroups, the prevalence of insulin resistance as defined by Matsuda ≤ 4 was 34%, 68% and 88%, by WHtR risk (WHtR > 0.5) 45%, 69% and 79% and by HTW risk 65%, 83% and 92%, respectively. The characteristics of the participants according to the three subgroups of the screening parameters WHtR and HTW phenotype are shown in Tables 2 and 3, respectively. The prevalences for the normal, the impaired/risk and the abnormal subgroups were 60%, 27% and 13% if assessed by fasting plasma glucose, 44%, 42% and 14% by WHtR and 23%, 54% and 23% by HTW phenotype. Metabolic and cardiovascular risk factors demonstrated better distinction between subgroups of increasing severity if grouping was based on phenotype parameters.

**Table 2:** Subgroups of waist-to-height ratio: differences between the groups are *p* < 0.001 unless indicated otherwise.

	Normal	Risk	Elevated WHtR
Total number (%)	986 (44.2)	940 (42.1)	297 (13.3)
Men (%)	592 (60.0)	395 (42.0)	196 (66.0) ns
Age	51 ± 15	59 ± 12	60 ± 13 *ns*
BMI (kg/m^2^)	24 ± 3	29 ± 3	35 ± 5
SBP (mmHg)	127 ± 17	136 ± 17	141 ± 18
Total cholesterol (mmol/l)	5.5 ± 1.1	5.5 ± 1.1 ns	5.5 ± 1.1 ns *ns*
LDL-C (mmol/l)	3.3 ± 1.0	3.4 ± 0.9 ns	3.4 ± 1.0 ns *ns*
Triglycerides (mmol/l)	1.3 ± 0.7	1.6 ± 1.3	1.8 ± 1.2 *#*
HDL-C (mmol/l)	1.6 ± 0.4	1.4 ± 0.4	1.3 ± 0.4 #
TG/HDL-C ratio	0.9 ± 0.8	1.3 ± 1.7	1.5 ± 1.3 *ns*
Fasting blood glucose (mmol/l)	5.4 ± 1.0	5.8 ± 1.3	6.6 ± 1.9
Fasting insulin (pmol/l)	61 ± 36	86 ± 48	123 ± 65
Matsuda index	6.0 ± 3.2	3.9 ± 2.4	2.8 ± 1.8
HTW class 0–2	0.60 ± 0.66	1.30 ± 0.47	1.43 ± 0.50 ##
Matsuda ≤ 4 (%)	28	63	80

Data are given as mean ± standard deviation. In comparison to normal ns = no significance, *p < .05 and **p < 0.01. In comparison to risk *ns* = *no significance, #p* < *0.05 and ##p* < *0.01.*

Normal, risk and elevated WHtR are defined in the method section. *SBP* systolic blood pressure, *HTW* classes as normal, risk and elevated are defined in the method section.

**Table 3:** Subgroups of hypertriglyceridemic-waist phenotype: differences between the groups are *p* < 0.001 unless indicated otherwise.

	Normal	Risk	Elevated HTW
Total number (%)	495 (23)	1189 (54)	509 (23)
Men (%)	347 (70)	571 (48)	290 (57) ##
Age	49 ± 16	58 ± 14	57 ± 12 *ns*
BMI (kg/m^2^)	23 ± 2	28 ± 4	30 ± 5
SBP (mmHg)	123 ± 16	134 ± 17	138 ± 19
Total cholesterol (mmol/l)	5.3 ± 1.1	5.4 ± 1.0 ns	5.9 ± 1.2
LDL-C (mmol/l)	3.1 ± 1.0	3.4 ± 0.9	3.5 ± 1.0 *ns*
Triglycerides (mmol/l)	0.9 ± 0.3	1.2 ± 0.5	2.7 ± 1.6
HDL-C (mmol/l)	1.8 ± 0.4	1.5 ± 0.4	1.3 ± 0.4
TG/HDL-C ratio	0.6 ± 0.3	.9 ± .6	2.4 ± 2.2
Fasting blood glucose (mmol/l)	5.2 ± 0.9	5.8 ± 1.3	6.3 ± 1.7
Fasting insulin (pmol/l)	52 ± 30	79 ± 46	108 ± 61
Matsuda index	6.9 ± 3.2	4.5 ± 2.7	3.0 ± 1.9
WHtR class 0–2	0.01 ± 0.09	0.81 ± 0.65	1.05 ± 0.66
Matsuda ≤ 4 (%)	13	52	80

Data are given as mean ± standard deviation. In comparison to normal ns = no significance, *p < .05 and **p < 0.01. In comparison to risk *ns* = *no significance, #p* < *0.05 and ##p* < *0.01.*

Normal, risk and elevated HTW are defined in the method section. *SBP* systolic blood pressure, *WHtR* classes as normal, risk and elevated are defined in the method section.

For all participants, the best inverse association was observed between the Matsuda index and the screening parameter WHtR (r = − 0.458) as depicted in Table 4, followed in descending order by waist circumference (r = − 0.445), by HTW phenotype (r = − 0.438), by BMI (r = − 0.404) and by the TG/HDL-C ratio. WHtR demonstrated similar correlation coefficients in the three subgroups as shown in Table 4. TG/HDL-C ratio did not correlate with the Matsuda index in the diabetes group. The correlation between the Matsuda index and FPG has a false high correlation coefficient due to the common factor FPG in both variables and, accordingly, cannot be used for comparison with the other markers. However, it allows the comparison for this marker FPG between the three subgroups. This correlation was highly significant in NFG (r = − 0.330), was weaker in IFG (r = − 0.182) and was lost in the diabetes group (r = − 0.080, n.s.).

**Table 4:** Correlation coefficients for the potential markers of dysmetabolic phenotype (*p* values are < 0.001 unless indicated otherwise).

		All	Normal FPG	IFG	Diabetes
nr		2233	1333	599	301
**Waist-Height ratio**	**Matsuda**	**− .458**	**− .396**	**− .402**	**− .391**
	Insulin	.433	.375	.344	.361
	FPG	.319	.208	*.124* xx	*.063* ns
	TG/HDL-C	.203	.264	*.102* x	*.031* ns
**HTW Phenotype**	**Matsuda**	**− .438**	**− .383**	**− .356**	**− .271** **xx**
	Insulin	.358	.343	.287	*.165* xx
**TG/HDL-C**	FPG	.259	.166 xx	.152 xx	***− .005*** ns
	**Matsuda**	**− .266**	**− .297**	**− .261**	***− .078***
	Insulin	.267	.305	.306	*.084* ns
	FPG	.309	.148	*.032*	*.105* .069
	Waist	.261	.361	*.182*	*.089* ns
	Waist-Height ratio	.203	.264	*.102* x	*.031* ns
**FPG**	Insulin	.337	.185	*.139*	*.072* ns
	TG/HDL	.309	.148	*.032* ns	.105 .069
	Waist	.316	.217	*.130* xx	*.066* ns
	Waist-Height ratio	.319	.208	*.124* xx	*.063* ns
	HTW Pheno	.259	.166 xx	.152 xx	*− .005* ns

xx = *p* < 0.01, *ns* = no significance.

Underlined numbers indicate relevant and similar associations in the three subgroups. Italicized numbers demonstrate a weak or lost correlation in one of the subgroups compared to NFG. Bold letters indicate the central correlations of a specific phenotype marker with the Matsuda index for comparison between these markers.

Discriminatory power was evaluated by areas under ROC curves as shown in Table 5. For the prediction of insulin resistance (Matsuda index ≤ 4), WHtR had the highest accuracy in the total group and in each of the three subgroups. There was an incremental decrease of this accuracy with HTW phenotype, TG/HDL-C and FPG.

**Table 5:** ROC area under the curve.

Insulin resistance	All	NFG	IFG	Diabetes
WHtR	.771	.758	.698	.780
HTW	.738	.740	.664	.735
TG/HDL-C	.729	.714	.650	.695
PG	.762	.673	.607	.483

*NFG* normal fasting glucose, *IFG* impaired fasting glucose.

**Figure 1:**
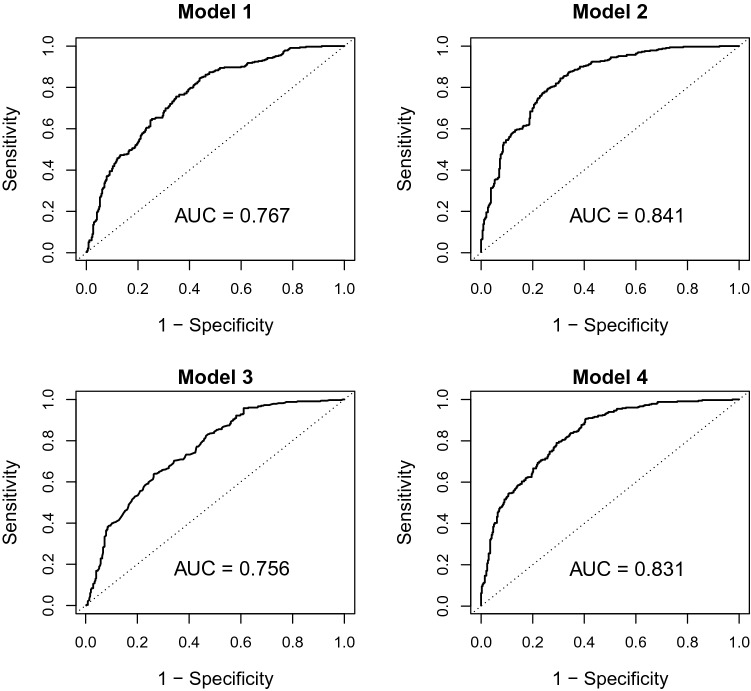
ROC curves for models 1–4 from the validation sample. Plots of the respective sensitivity against the false positive rate (1 minus specificity). The area under the curve (AUC) of an ideal binary classifier is 1, the AUC of a test without discriminatory power is 0.5.

**Figure 2:**
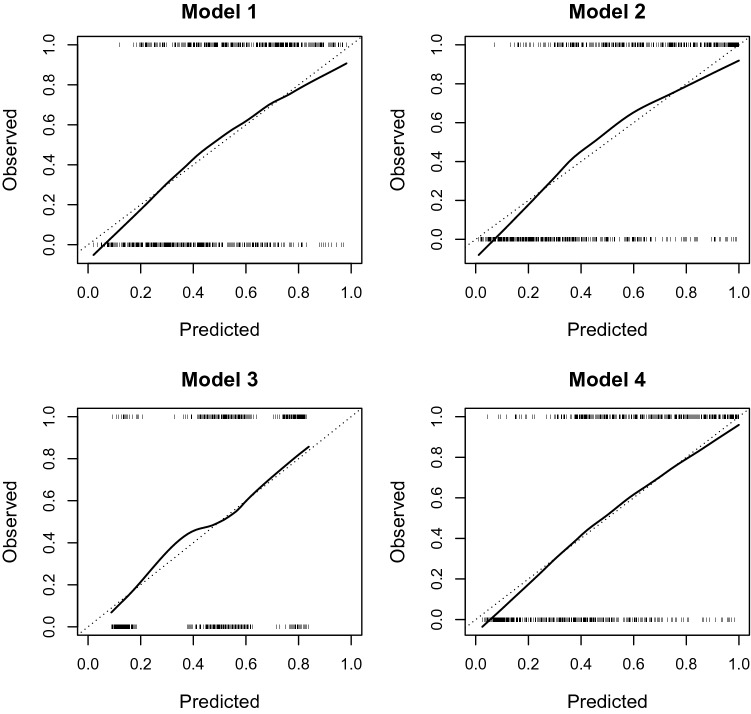
Calibration curves for models 1–4 from the validation sample. Plots of observed frequencies of insulin resistance (1 or 0) against predicted probabilities from the respective models. The ideal calibration curve is the bisector of the coordinate system (dashed line).

Tables 6 and 7 depict regression coefficients from models predicting insulin resistance. Models 1 and 2 use WHtR and its interaction with sex as predictor, models 3 and 4 are based on HTW phenotype. Models 1 and 3 control for age and sex, models 2 and 4 include FPG and TG/HDL-C. The regression coefficients and the individual variable values can be combined to yield an individual linear predictor *η*_*i*_. Higher values indicate higher individual risks. Individual probabilities can be calculated as *p*_*i*_ = exp(*η*_*i*_)/[1 + exp(*η*_*i*_)], where exp denotes the standard exponential function to the base e^32^. Figures 1 and 2 depict the results of the validation of these models in the internal validation sample in terms of discriminatory power (ROC curves in Fig. 1) and calibration (calibration curves in Fig. 2)^33^.

**Table 6:** Firth logistic regression models for the prediction of insulin resistance (Matsuda index < 4) using WHtR.

Variable	Regression coefficient	Standard error	*p* value
**Model 1**			
Intercept	− 7.7980	0.7030	< .0001
Age (per year)	0.00226	0.00463	0.6255
Male sex	− 3.1672	1.2916	0.0142
WHtR (per unit)	13.3820	1.2607	< .0001
WHtR × male	6.3336	2.3113	0.0061
**Model 2**			
Intercept	− 11.4816	0.8621	< .0001
Age (per year)	− 0.00219	0.00509	0.6663
Male sex	− 4.1626	1.3930	0.0028
WHtR (per unit)	10.4148	1.3112	< .0001
WHtR × male	6.8426	2.4735	0.0057
Fasting blood glucose (per mmol/l)	0.9109	0.1020	< .0001
TG/HDL-C (per mmol/l)	0.8678	0.1144	< .0001

**Table 7:** Firth logistic regression models for the prediction of insulin resistance (Matsuda index < 4) using HTW phenotype.

Variable	Regression coefficient	Standard error	*p* value
**Model 3**			
Intercept	− 2.5594	0.2855	< .0001
Age (per year)	0.0119	0.00448	0.0078
Male sex	0.2359	0.1244	0.0579
HTW Phenotype			< .0001
0 (reference)	0		
1	1.5302	0.3220	< .0001
2	1.8433	0.1886	< .0001
3	3.1022	0.2198	< .0001
**Model 4**			
Intercept	− 7.5758	0.5822	< .0001
Age (per year)	0.00457	0.00483	0.3440
Male sex	− 0.4244	0.1527	0.0054
HTW Phenotype			< .0001
0 (reference)	0		
1	0.6977	0.3712	0.0602
2	1.6286	0.1968	< .0001
3	2.0175	0.2786	< .0001
Fasting blood glucose (per mmol/l)	0.9846	0.1016	< .0001
TG/HDL-C (per mmol/l)	0.6806	0.1483	< .0001

## Discussion

Prediabetes and diabetes describe a range of heterogeneous metabolic states with varying degrees of insulin resistance and beta-cell dysfunction^34^. While acknowledging that T2D on a molecular and cellular level is of high complexity^4^, its clinical hallmark is the presence of insulin resistance and/or compensatory hyperinsulinemia, conditions that are associated with atherosclerotic cardiovascular disease^12,15^ and heart failure with preserved ejection fraction^35^. Insulin resistance commonly precedes the presence of hyperglycemia for years or even decades^36^. These metabolic traits often occur in a distinct metabolic phenotype with visceral adiposity, elevated triglycerides, low HDL-C, and elevated blood pressure, a constellation that has been termed metabolic syndrome or Syndrome X respectively^36^. There are numerous signals indicative of this constellation of metabolic traits, long before changes in glycemic markers become evident. In this regard, the combination of anthropometric and biochemical markers indicative of visceral adiposity, which can be detected on clinical examination and on standard biomarker panels, hold promise in detecting metabolically deranged individuals at high cardiometabolic risk.

The present analysis offers insight into the association between anthropometric and biochemical markers indicative of metabolic dysregulation and the Matsuda index in a large representative group of Caucasians.

*Waist-to-height ratio (WHtR)* is an easy to obtain and inexpensive metric indicative of the metabolic phenotype at high risk for cardiovascular events. It has been shown to be superior to BMI and/or waist circumference alone. Meta-analytic evidence has shown WHtR to be a significantly better predictor than waist circumference for diabetes, cardiovascular disease and all-cause mortality. The suggested general cutoff is 0.5^18,19^. In the present analysis, WHtR showed the best association with the Matsuda index also in all subgroups. This may prove particularly beneficial both in the prediabetic and the overt diabetic stages. In individuals with early stages of diabetes, insulin resistance/compensatory hyperinsulinemia is a major risk factor for complications but may be underdiagnosed by the use of FPG in clinical routine settings. This may result in missed opportunities for diabetes prevention and/or early intervention. In individuals with overt diabetes, persistent insulin resistance/hyperinsulinemia remains a major risk for complications and is even more difficult to assess due to the unknown degree of betacell dysfunction. These complications, however, cause a lot of harm to the affected individuals and financially to society. In this regard, the data from our study suggest that WHtR may constitute a useful and robust surrogate for the diagnosis of insulin resistance and/or compensatory hyperinsulinemia. The discriminatory power for the prediction of insulin resistance is fair (area under the ROC curve 0.765) when taking only age, sex and WHtR into account, i.e. measures which are widely available diagnostic tools. A good predictive power (area under the ROC curve 0.841) is suggested, when adding also FPG and TG/HDL-C into the predictive model, measures that are more commonly available in central European outpatient clinics and primary care facilities.

*Hypertriglyceridemic Waist* Elevated waist circumference is predictive of visceral adiposity at any given BMI^15^. Visceral adiposity is closely intercorrelated with hepatic fat infiltration (NAFLD), increased hepatic very-low-density lipoprotein (VLDL) production, and hypertriglyceridemia^37,38^. When using waist circumference as a surrogate, increased visceral fat is an independent risk factor for high-risk atherosclerosis^13,39^ and coronary artery disease and death^40^ and triglyceride-related risk has been suggested to be causal in atherosclerotic cardiovascular disease^41^. Hypertriglyceridemic waist (HTW), a visceral adiposity marker combining elevated waist circumference (≥ 90 cm in men) and elevated fasting plasma triglycerides (≥ 2 mmol/L), is thus indicative of the high-risk cardiometabolic phenotype^20^ and/or high-risk atherosclerosis^42^. We have demonstrated in an apparently healthy Caucasian population^28^ that the HTW phenotype has a fair diagnostic accuracy in the prediction of the concomitant presence of insulin resistance, dyslipidemia and the metabolic syndrome (area under the curve 0.773). Diagnostic accuracy was similar in this study (area under the curve 0.781) extending these findings to a larger cohort (n = 2231) comprising participants with normal fasting glucose (n = 1333), impaired fasting glucose (n = 599) and with T2DM (n = 299). In this analysis, the HTW phenotype was the second-best screening marker associated with the Matsuda index also in all subgroups. Similarly, in the predictive models, the HTW phenotype demonstrated good accuracy with an area under the ROC curve of 0.756 for model 3 and 0.831 for model 4.

*Triglycerides-to-HDL-C ratio (TG/HDL-C)* is a metabolic index that can be derived from standard lipid profile. It is associated with both insulin resistance and closely reflects the lipoprotein pattern referred to as atherogenic lipoprotein phenotype^43^, including the predominance of a small LDL phenotype^22^. Furthermore, a higher TG/HDL-C has been linked to higher prevalence of thin-cap fibroatheromas in coronary artery disease^24–26^. This is of relevance because individuals living with diabetes are considered to be at high risk for adverse cardiovascular events, which is the leading cause of death in this subgroup^40^. Varying cutoff points of TG/HDL-C ratio have been proposed in the literature. We have demonstrated that the TG/HDL-C has a good diagnostic accuracy in the prediction of the concomitant presence of insulin resistance, dyslipidemia and the metabolic syndrome (area under the ROC curve 0.817) with optimal cut-off points of 1.22 for men (80% sensitivity, 71% specificity) and 0.83 for women (80% sensitivity, 75% specificity)^28^. The present study with extension to a larger cohort (n = 2231) comprising participants with normal fasting glucose (n = 1333), impaired fasting glucose (n = 599) and with T2DM (n = 299) showed a weak inverse association of TG/HDL-C with the Matsuda index for the total group and in the NPG and IFG subgroups. In individuals with diabetes, this association was no longer evident.

*Fasting plasma glucose (FPG)* has limitations as an early indicator of T2D. Of concern, relying on FPG in preventive care might fail to detect a substantial subgroup with insulin resistance. In our analysis, 34% of individuals classified as healthy by means of FPG (NFG subgroup) had a pathological Matsuda index. These findings are of concern since they imply that risk stratification based on FPG may misclassify up to one third of individuals with pathological insulin sensitivity as healthy and subsequently may result in missed chances for prevention^12^. As by definition, FPG does not identify individuals with isolated impaired glucose tolerance. Furthermore, FPG is being mainly driven by cortisol levels, and thus may result in a high rate of false positives (i.e. low specificity). In our study, the association of FPG with the metabolic state of insulin resistance was compared in the three subgroups. Interestingly, the significant association in the NFG group was weak in the IFG group and was lost in the diabetes group. Most likely, the increasing betacell dysfunction in the course of T2D contributes to this effect. This corroborates the important message that FPG measurements may not fully depict the high-risk metabolic state due to insulin resistance and compensatory hyperinsulinemia in individuals with diabetes. Clinically though, the avoidance of diabetic complications triggered by insulin resistance/hyperinsulinemia remains a major task. Our study data suggest that WHtR may be a more robust screening parameter in this respect.

### Strengths and limitations

These data offer insights into the association between anthropometric and biochemical markers indicative of metabolic dysregulation and the Matsuda index resulting in two models for the prediction of insulin resistance. This could serve as an adjunct and/or if not available replace the assessment of indices derived from combined measurements of glucose/insulin serum levels such as HOMA-IR or Matsuda Index in clinical care.

A major strength of this study is the large sample and the presence of a 5-point OGTT including measures of glucose and insulin at all time-points, which is the best clinically available tool for assessing insulin sensitivity/resistance. This direct and time-consuming measurement of insulin sensitivity is limited to clinical studies, not used in day-to-day clinical care and has been suggested as the closest surrogate for the euglycemic insulin clamp technique. For this study, we calculated the Matsuda index, which allowed us to empirically test its association with potentially easy, non-expensive and widely available clinical biomarkers and anthropometric markers for impaired glucose metabolism and/or risk for cardiovascular disease.

However, our data are not without limitations. First, the cross-sectional nature of this study design limits our ability to establish causal relationships or to determine the directionality of the observed effects. Second, this analysis presents data from a Caucasian population and therefore limits the generalization of these results for different ethnicities as ethnicity impacts on the TG/HDL-C ratio. In African Americans, triglycerides and the TG/HDL-C ratio do not reliably predict IR. This has been linked to the observation that insulin resistance does not impair lipoprotein lipase in this subgroup and thus does not induce hypertriglyceridemia^44^.

## Conclusion

In conclusion, the WHtR showed the best predictive value for insulin resistance, including in the subgroups with impaired fasting glucose and with T2D. This anthropometric marker could thus serve as an adjunct marker for detecting insulin resistance and compensatory hyperinsulinemia in primary care settings where more extended testing such as postprandial glucose and insulin assays are not available. Clinical markers with higher predictive value for early stages of diabetes than fasting glucose could refine phenotypic screening and might offer potential to ameliorate early identification of individuals who are candidates for appropriate therapeutic interventions aimed at prevention of diabetes and/or of diabetic complications and cardiovascular disease. Insulin resistance and compensatory hyperinsulinemia can be efficaciously reversed by lifestyle modification^45^. Accordingly, improved early identification of insulin resistance may represent a first step towards better preventive care aiming to address the massive economic and societal burden of diabetes worldwide.
